# Accelerometry assessed physical activity of older adults hospitalized with acute medical illness - an observational study

**DOI:** 10.1186/s12877-020-01763-w

**Published:** 2020-10-02

**Authors:** Plamena Tasheva, Vanessa Kraege, Peter Vollenweider, Guillaume Roulet, Marie Méan, Pedro Marques-Vidal

**Affiliations:** Department of medicine, internal medicine, Lausanne university hospital and University of Lausanne, Rue du Bugnon 46, 1011 Lausanne, Switzerland

**Keywords:** Physical activity, Aged, Aged, 80 and over, Internal medicine, Observational study

## Abstract

**Background:**

In a hospital setting and among older patients, inactivity and bedrest are associated with a wide range of negative outcomes such as functional decline, increased risk of falls, longer hospitalization and institutionalization. Our aim was to assess the distribution, determinants and predictors of physical activity (PA) levels using wrist-worn accelerometers in older patients hospitalized with acute medical illness.

**Methods:**

Observational study conducted from February to November 2018 at an acute internal medicine unit in the University hospital of Lausanne, Switzerland. We enrolled 177 patients aged ≥65 years, able to walk prior to admission. PA during acute hospital stay was continuously recorded via a 3D wrist accelerometer. Clinical data was collected from medical records or by interview. Autonomy level prior to inclusion was assessed using Barthel Index score. PA levels were defined as < 30 mg for inactivity, 30–99 mg for light and ≥ 100 for moderate PA. Physically active patients were defined as 1) being in the highest quartile of time spent in light and moderate PA or 2) spending ≥20 min/day in moderate PA.

**Results:**

Median [interquartile range - IQR] age was 83 [74–87] years and 60% of participants were male. The median [IQR] time spent inactive and in light PA was 613 [518–663] and 63 [30–97] minutes/day, respectively. PA peaked between 8 and 10 am, at 12 am and at 6 pm. Less than 10% of patients were considered physically active according to definition 2. For both definitions, active patients had a lower prevalence of walking aids and a lower dependency level according to Barthel Index score. For definition 1, use of medical equipment was associated with a 70% reduction in the likelihood of being active: odds ratio (OR) 0.30 [0.10–0.92] *p* = 0.034; for definition 2, use of walking aids was associated with a 75% reduction in the likelihood of being active: OR = 0.24 [0.06–0.89], *p* = 0.032.

**Conclusion:**

Older hospitalized patients are physically active only 10% of daily time and concentrate their PA around eating periods. Whether a Barthel Index below 95 prior to admission may be used to identify patients at risk of inactivity during hospital stay remains to be proven.

## Background

Extended bedrest has been described as toxic for older patients [1]. In a hospital setting and among older hospitalized patients, inactivity and bedrest are associated with a wide range of negative outcomes such as functional decline, increased risk of falls, longer hospitalization and new institutionalization [2–5]. Assessment of PA relies mostly on qualitative nursing observation [6] and remains poorly documented in hospital electronic records [7]. Therefore, there is an urging need to objectively monitor and quantify inpatient PA levels.

Most PA levels are assessed based on observational data such as periodic nursing reports [2, 7, 8] or on nurse standardized functional assessment [6, 9]. These qualitative assessments are less accurate compared to objective measures of PA such as accelerometry [10]. Indeed, accelerometers allow the collection of objective and continuous PA data and have been tested and validated in older patients [11–14]. Further, the data collected can be analyzed with algorithms that classify locomotion and non-locomotion periods in everyday life [15]. Still, there is a paucity of studies assessing PA levels by accelerometry in hospitalized older patients [12–14, 16–22]. In a previous paper, Lim et al. reported that the PA levels of 38 hospitalized older patients were very low and that most PA was sustained over short periods [12]. However, the sample size was small, and the results were not replicated in other settings.

Therefore, the aims of this study were to assess the distribution, the determinants of PA levels and the variables allowing identification of older hospitalized patients at risk of physical inactivity, by means of a wrist-worn accelerometer.

## Methods

### Setting

We conducted this study from February 2018 to November 2018 in a 21-bed internal medicine ward of the Lausanne university hospital (CHUV), in canton Vaud, in the French speaking part of Switzerland. The CHUV has over 1500 beds and admits over 50,000 patients per year.

### Recruitment

Patients were recruited on a daily basis, from Monday to Friday. All patients aged ≥65 years admitted directly to the study ward or via the emergency unit were considered eligible. Participants were excluded if they: a) had a probable life expectancy of less than 30 days, based on clinical judgment; b) had insufficient comprehension of French language, c) were unable to stand within the week before hospitalization, as assessed by interview, or d) were forced to bedrest by factors not directly related to the disease (e.g. fracture). The selection procedure was applied within the first three days of hospitalization. If exclusion criteria were not met, patients were invited to participate and received an explanation of the study procedure. If the patient accepted, a written informed consent was signed before the start of the study.

All investigators had previously been trained regarding screening and recruiting methods.

### Ethical statement

The study was approved by the Swiss Ethics Committee on research involving humans using BASEC (www.cer-vd.ch), reference 2017–01907 (decision of 21 December 2017). The full decision of the CER-VD can be obtained from the authors upon request. The study was performed in agreement with the Helsinki declaration and its former amendments, and in accordance with the applicable Swiss legislation. All participants or their legal representatives (in case of confusion or cognitive impairment) provided a signed informed consent before entering the study. If a participant decided to withdraw from the study, data collected until the moment of withdrawal was used.

### Physical activity assessment

We assessed PA levels using a wrist accelerometer (GENEActiv Original, ActivInsights Ltd., UK), parametrized at 50 Hz. These accelerometers have been shown to provide a reliable and valid measurement of physical activity in adults [23] and hospitalized older patients [12]. We provided the patients with a device immediately after inclusion and they could choose on which wrist to wear it. Previous studies have shown that wrist side does not influence measurements [24]. Patients were asked to wear the device continuously (day and night, including showering). The observation period was limited to the index hospitalization in internal medicine. Upon discharge or transfer to another department (for e.g. intensive care, surgery), the accelerometer was removed by a nurse or one of the investigators.

Accelerometry data was extracted and analyzed using version 9.1 the GGIR package for R [23]. This package estimates time spent in different levels of PA according to predefined thresholds, overall and separately for day and night (R-script provided in annex 2). A valid day was defined as at least 16 h of daytime wear. Moreover, at least 24-h of valid data were required for analysis [25]. PA levels were defined using the thresholds proposed by Bakrania et al. [26]: < 30 mg for inactivity, 30–99 mg for light, and ≥ 100 for moderate PA. Of note, no patient had vigorous PA. As no valid criteria have been set to define a patient as being active, two definitions were applied: 1) being in the highest quartile of time spent in light and moderate PA, and 2) spending at least 20 min/day in moderate PA.

### Covariates

Investigators extracted covariates from the hospital electronic database. This included demographics; reason for hospitalization; comorbidities via the Charlson comorbidity index [27]; presence of cognitive impairment/confusion based on medical documentation upon inclusion, use of sedative drugs at admission; prescription (yes/no) of physiotherapy. During the baseline interview, investigators collected self-reported physical function 2 weeks before admission (i.e. use of walking aids and history of falls during the year before admission), medical equipment upon inclusion (i.e. urinary catheter or oxygen therapy) and isolation precautions (i.e. for infection control and patient protection).

Prior research suggests that admissions for gait problems/fall, general state of health alteration, and neurological deficit, are predictors of functional decline in hospitalized older patients [28, 29]. Hence, for our analysis, we created a variable based on these conditions, also including musculoskeletal pain, and named it “reason for admission associated with functional decline”.

Autonomy prior to hospital admission was assessed using the Barthel Index score, reported as being the best scale to assess activities of daily living (ADL) [30] and with a widespread use. The modified version improves the internal consistency and provides better discrimination of functional ability. For patients with cognitive impairment or confusion, the level of autonomy before hospitalization was assessed by interviewing their relatives or caregivers, in face-to-face interviews or by phone call. The patient’s ability to perform different ADLs was rated as follows: fully independent; with minimal or moderate help; attempts task but unsafe; and unable to perform. Maximum score was 100. A total Barthel Index score of 0–20 suggests total, 21–60 severe, 61–90 moderate and 91–99 slight dependence. A score of 100 indicates that the patient is independent of assistance from others.

Skin status and risk of bedsores was assessed using the Braden score upon inclusion [31]. The Braden scale rates patients using six subscales: sensory perception, moisture, activity, mobility, nutrition, and friction and shear. The maximum score is 23; a score ≤ 18 indicates a high risk of sore development.

### Statistical analysis

Statistical analysis was conducted using Stata v15.1 (Stata Corp, College Station, TX, USA). Results are expressed as number of patients and (percentage) for categorical variables and as average ± standard deviation or as median [interquartile range] for continuous variables. Between-group comparisons were performed using chi-square or Fisher’s exact test for categorical variables and analysis of variance or Kruskal-Wallis nonparametric test for continuous variables. Variables significantly and independently associated with PA status were identified by stepwise forward logistic regression, using the category physically active coded as a binary (0/1) variable, and a *p*-value for entry of 0.05. Variables significantly associated with physical activity in the bivariate analysis were included and the results of the logistic regression were expressed as odds ratio (OR) and 95% confidence interval (CI). The screening capacity of the Barthel index to identify active patients according to the different definitions was assessed by identifying the optimal threshold using the **cutpt** command of Stata. For each threshold, we computed the resulting area under the receiver-operating curve (AROC), sensitivity, specificity, positive and negative predictive values and corresponding 95% confidence intervals.

Sensitivity analyses were conducted as follows: a) after excluding participants with over 20% accelerometer non-wear time; b) using two other definitions of being active: 1) the highest tertile or 2) the highest quintile of time spent in light and moderate PA. Statistical significance was assessed for a two-sided test with a *p*-value < 0.05.

## Results

### Sample selection

Of the 377 patients screened, 274 were eligible for the study and invited to participate. Among the 274 eligible patients, 211 (77%) accepted to participate, of whom 177 (84%) had valid accelerometry data. The selection procedure is summarized in Supplemental Fig. 1. The characteristics of eligible patients who accepted versus who did not accept to participate are summarized in Supplemental Table 1. Women participated less frequently, while no differences were found for the other characteristics.

The characteristics of the participants according to gender are summarized in Table 1. Women were significantly older, had a lower BMI, a lower Charlson comorbidity index and a lower frequency of cognitive impairment than men, while no differences were found for all other characteristics.

**Table 1:** baseline characteristics of patients according to sex, NEXT-STEP study, Lausanne, Switzerland

	Men	Women	***P***-value
Number of patients	106	71	
Characteristics			
Age (years)	79.7 ± 8.1	83.5 ± 8.6	0.003
Body mass index (kg/m^2^) ^a^	25.3 ± 4.6	23.7 ± 4.5	0.039
Depressive disorders	12 (11.3)	14 (19.7)	0.122
Urinary/fecal incontinence	41 (38.7)	19 (26.8)	0.101
Hearing loss/vision issues	45 (42.5)	25 (35.2)	0.334
Medical history			
Walking aids 2 weeks before admission	49 (46.2)	41 (57.8)	0.133
History of falls during the year before admission	31 (29.3)	23 (32.4)	0.656
Reason for admission associated with functional decline ^b^	59 (55.7)	34 (47.9)	0.310
Status upon inclusion			
Cognitive impairment/confusion	37 (34.9)	15 (21.1)	0.049
Sedative drugs	15 (14.2)	12 (17.1)	0.590
Barthel Index	89.1 ± 15.4	88.6 ± 16.4	0.854
Braden score			0.566
> 18	55 (54.5)	35 (50.0)	
≤ 18	46 (45.5)	35 (50.0)	
Medical equipment ^c^	26 (24.5)	10 (14.1)	0.091
Isolation precautions	3 (2.8)	3 (4.2)	0.685‡
Prescription of physiotherapy	66 (62.3)	47 (66.2)	0.593
Charlson comorbidity index	4 [3–7]	3 [1–6]	0.002¶

Results are expressed as mean ± SD or as median [interquartile range] for continuous variables and as number of participants (percentage) for categorical variables. ^a^, 99 men and 63 women. ^b^: gait problems/ fall, general state alteration, musculoskeletal pain, neurological deficit. ^c^: urinary catheter or nasal cannula oxygen therapy. Between-group comparisons using student’s t-test or Kruskal-Wallis (¶) test for continuous variables and chi-square or Fisher’s exact test (‡) for categorical variables

### Physical activity levels and distribution throughout the day

**Fig. 1:**
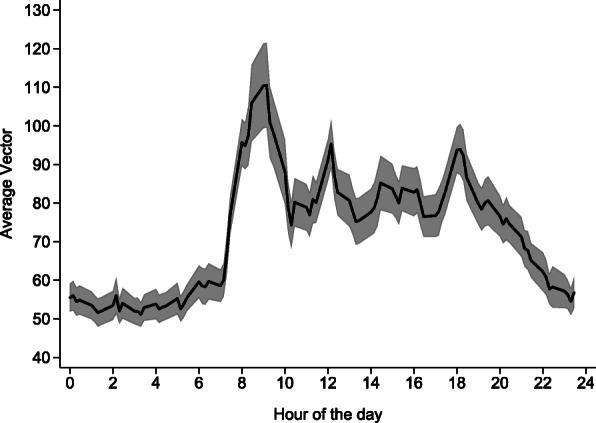
average physical activity per hour among hospitalized older patients, NEXT-STEP study, Lausanne, Switzerland. Results are shown as average and 95% confidence interval

Over 3700 h of PA time were recorded. Examples of PA recordings for a physically active and inactive patient are provided in supplemental Figs. 2 and 3. PA levels overall and according to gender are summarized in Table 2. Overall, within eleven hours of accelerometer-based monitoring (nighttime not included), patients were inactive ten hours per day and active one hour per day (10%). There was no difference between genders. PA distribution according to the period of the day is represented in Fig. 1. Peaks of PA were found between 8 and 10 am, at 12 am and at 6 pm.

**Table 2:** Physical activity during the day according to gender, NEXT-STEP study, Lausanne, Switzerland

N	All	Men	Women	***P***-value
	177	106	71	
Inactivity				
Minutes / day	613 [518–663]	618 [518–661]	605 [503–666]	0.842
% of daily time	90.4 [84.8–94.3]	90.6 [85.2–94.3]	90.1 [84.3–94.2]	0.732
Light physical activity				
Minutes / day	63 [30–97]	58 [32–95]	70 [27–103]	0.798
% of daily time	9.1 [5.5–14.1]	8.9 [5.3–13.4]	9.7 [5.5–14.6]	0.687
Moderate physical activity				
Minutes / day	2 [1–9]	2 [1–9]	3 [1–9]	0.795
% of daily time	0.4 [0.1–1.2]	0.4 [0.2–1.2]	0.4 [0.1–1.4]	0.937

Results are expressed as median [interquartile range]. Between-group comparisons using Kruskal-Wallis test

### Physically active versus inactive patients

Less than 10% of patients were considered as physically active using definition 2 (≥20 min/day of moderate PA). The characteristics of physically active and inactive patients are summarized in Table 3.

**Table 3:** Baseline characteristics of hospitalized older patients according to physical activity levels, NEXT-STEP study, Lausanne, Switzerland. Physically active subjects were defined as being in the highest quartile of time spent in light and moderate PA (definition 1) or as spending at least 20 min of moderate physical activity per day (definition 2)

Number of patients	Definition 1			Definition 2		
	Inactive	Active	***P***-value	Inactive	Active	***P***-value
	132	45		163	14	
**Characteristics**						
Women	52 (39.4)	19 (42.2)	0.738	65 (39.9)	6 (42.9)	0.827
Age (years)	81.7 ± 8.5	80.0 ± 8.3	0.261	81.4 ± 8.6	79.3 ± 6.9	0.367
Body mass index (kg/m^2^) ^a^	24.7 ± 4.7	24.4 ± 4.6	0.695	24.8 ± 4.7	23.2 ± 3.5	0.269
Depressive disorders	21 (15.9)	5 (11.1)	0.432	24 (14.7)	2 (14.3)	1.000‡
Urinary/fecal incontinence	45 (34.1)	15 (33.3)	0.926	56 (34.4)	4 (28.6)	0.775‡
Hearing loss/vision issues	51 (38.6)	19 (42.2)	0.671	64 (39.3)	6 (42.9)	0.792
**Anamnesis**						
Walking aids 2 weeks before admission	73 (55.3)	17 (37.8)	0.042	87 (53.4)	3 (21.4)	0.026‡
History of falls during the year before admission	40 (30.3)	14 (31.1)	0.919	52 (31.9)	2 (14.3)	0.232
Reason for admission associated with functional decline ^b^	74 (56.1)	19 (42.2)	0.108	90 (55.2)	3 (21.4)	0.023‡
**Status upon inclusion**						
Cognitive impairment/confusion	40 (30.3)	12 (26.7)	0.644	52 (31.9)	0 (0)	0.011‡
Sedative drugs	18 (13.7)	9 (20.0)	0.315	25 (15.4)	2 (14.3)	1.000
Barthel Index	87.5 ± 16.7	93.2 ± 12.1	0.037	88.2 ± 16.2	97.4 ± 3.7	0.035
Braden score			0.133			0.362
> 18	62 (49.2)	28 (62.2)		81 (51.6)	9 (64.3)	
≤ 18	64 (50.8)	17 (37.8)		76 (48.4)	5 (35.7)	
Medical equipment ^c^	32 (24.2)	4 (8.9)	0.027	34 (20.9)	2 (14.3)	0.738‡
Isolation precautions	3 (2.3)	3 (6.7)	0.173‡	5 (3.1)	1 (7.1)	0.395‡
Prescription of physiotherapy	89 (67.4)	24 (53.3)	0.089	107 (65.6)	6 (42.9)	0.089
Charlson comorbidity index	4 [2–6]	4 [2–6]	0.370¶	4 [2–6]	3 [2–7]	0.406¶
Number of comorbidities	2 [1–4]	2 [1–3]	0.326¶	2 [1–4]	2 [1–3]	0.343¶

Results are expressed as mean ± SD or as median [interquartile range] for continuous variables and as number of participants (percentage) for categorical variables. ^a^, 99 men and 63 women. ^b^: gait problems/ fall, general state alteration, musculoskeletal pain, neurological deficit. ^c^: urinary catheter or oxygen therapy. Between-group comparisons using student’s t-test or Kruskal-Wallis test (¶) for continuous variables and chi-square or Fisher’s exact test (‡) for categorical variable

For both definitions, physically active patients had a lower prevalence of walking aids use 2 weeks before admission, and had a lower dependency level according to the Barthel Index score. Further, physically active patients according to definition 1 less frequently had a urinary catheter or nasal cannula oxygen therapy, while physically active patients according to definition 2 were less frequently admitted for a reason associated with functional decline and presented less frequently with cognitive impairment/confusion. No difference in PA was noted according to gender.

For definition 1, use of medical equipment was associated with a 70% reduction in the likelihood of being active: odds ratio 0.30 [0.10–0.92] *p* = 0.034; for definition 2, use of walking aids 2 weeks before admission was associated with a 75% reduction in the likelihood of being active: odds ratio 0.24 [0.06–0.89], *p* = 0.032.

For both definitions of physically active patients, the optimal threshold for the Barthel Index to detect a physically active patient was 95, leading to moderate sensitivity and specificity (Table 4).

**Table 4:** screening capacity of the Barthel index to identify active patients

Definition	Threshold	AROC	Sensitivity (%)	Specificity (%)	PPV (%)	NPV (%)
1	95	0.595 (0.512–0.678)	64.4 (48.8–78.1)	54.5 (45.7–63.2)	32.6 (23.0–43.3)	81.8 (72.2–89.2)
2	95	0.692 (0.590–0.795)	85.7 (57.2–98.2)	52.8 (44.8–60.6)	13.5 (7.2–22.4)	97.7 (92.0–99.7)
3	95	0.581 (0.503–0.658)	61.0 (47.4–73.5)	55.1 (45.7–64.3)	40.4 (30.2–51.4)	73.9 (63.4–82.7)
4	96	0.642 (0.554–0.730)	66.7 (49.0–81.4)	61.7 (53.1–69.8)	30.8 (20.8–42.2)	87.9 (79.8–93.6)

Results are expressed as value (95% confidence interval). AROC, area under the receiver operating curve; NPV, negative predictive value; PPV, positive predictive value. Definition 1: highest quartile of time spent in non-sedentary activities; definition 2, spending at least 20 min of moderate physical activity per day; definition 3, highest tertile of time spent in non-sedentary activities, definition 4, highest quintile of time spent in non-sedentary activities

Similar findings were obtained when analyses were restricted to patients with less than 20% of accelerometer non-wear time (supplemental Tables 2 and 3) or when other definitions of being physically active were used (supplemental Table 4).

## Discussion

There is little information regarding physical activity of hospitalized patients. To our knowledge, this is one of the largest studies measuring physical activity by accelerometry in older patients hospitalized with acute medical illness. According to our results, older hospitalized patients are inactive most of the time, and their PA is distributed into daily patterns.

### Physical activity levels and distribution throughout the day

Patients spent a median of approximately 1 h/day on PA, a value lower than the 4.2 h/day reported by Lim et al.’s study [12], who analyzed locomotion in 38 acutely hospitalized older patients (median age of 87.8 years). Conversely, another study reported only 43 min per day spent standing or walking [32] in a sample of 45 hospitalized older patients (median age 74.2 years) capable of walking independently pre-admission. Another study also reported that 30 older patients (median age 83.6 years) spent less than an hour between 9 am and 5 pm in an upright position and nearly 50% of the day lying down [19]. Finally, a study reported even lower durations spent walking (7 min/day) and standing up (35 min/day) in 100 older patients (median age 84 years) [17].

Interstudy comparison and reproducibility is very difficult because of the use of different thresholds and different PA metrics. Older hospitalized patients are characterized by very low PA levels, and thresholds to define PA in this population are rare and differ according to studies. In our study, PA levels were defined according to Bakrania et al. [26], light PA being defined by an acceleration ≥ 30 mg, whereas in the study of Lim et al. [12], a 1-min mean acceleration ≥ 12 mg was selected to define PA. Thresholds similar to those proposed by Lim et al. have been reported in free-living older people (median age ≥ 65 years) [13, 33] and younger [26, 34] populations. Still, other thresholds for light PA, developed and validated in laboratory calibration or in free-living populations, are usually ≥40 mg [35–38]. Analysis of our recordings with a thresholds of ≥85 mg as proposed by White et al. [35], ≥30 mg as proposed by Bakrania et al. [26], and ≥ 12 mg as proposed by Lim et al. [12] led to PA levels of 6, 65 and 175 min/day, respectively (supplementary Table 4). Hence, further studies are necessary to define the threshold that correctly identifies sedentary hospitalized patients at risk of complications.

The distribution of PA according to the period of day showed peaks between 8 and 10 am, at 12 am and at 6 pm. Our findings replicate Lim et al.’s observations [12] in a larger sample and suggest that older inpatients mobilize primarily during meal (eating) periods.

### Physically active versus inactive patients

Compared to physically active patients, physically inactive patients more frequently reported the use of walking aids 2 weeks before hospitalization, were more frequently admitted for a reason associated with functional decline, and had a higher dependency level according to Barthel Index score. These findings are consistent with other studies [8, 39, 40]. Moreover, our results suggest that initial evaluation of patients using these metrics could help to identify patients in need of mobilization during hospital stay [9].

Our results suggest that increased efforts are necessary to mobilize hospitalized patients. Nevertheless, the magnitude of efforts needed to achieve an adequate amount of PA during hospitalization may exceed the existing resources of the hospital. Future studies should try to estimate not only the minimum amount of PA needed to prevent increased in-hospital morbidity or length of stay, but also the optimal conditions necessary to deliver mobility interventions.

Physically active patients were less likely to have cognitive impairment/confusion. A possible explanation is that patients with declining cognitive function reduce their PA in an unknown environment. Our findings are in agreement with a Danish study including 48 older patients (mean age 84 years), where cognitive impairment at admission was associated with lower PA levels during hospitalization [41]. Conversely, a Norwegian study including 38 older patients (median age 83 years) found no association between cognitive impairment and PA [16]. Indeed, most studies assessing PA in older hospitalized adults excluded patients with cognitive impairment [11, 12, 39, 42, 43]. Overall, our results suggest that greater PA levels might be associated with a lower risk of cognitive impairment/confusion, a finding in agreement with recommendations for preventing this status [44].

### Implications for clinical practice

Our results strengthen the available evidence that hospitalized older patients move very little and that some indicators such as the Barthel Index assessed at admission are relevant to identify patients at risk of inactivity during hospital stay. An interesting finding were the peaks of PA during mealtimes, also reported in another study [12]. Hence, a possible way to favor patients’ locomotion would be to serve meals in common rooms instead of in hospital beds. Other alternatives include mobilization by family members or volunteers (if the patient’s condition allows it) to compensate for lack of resources [45]. This alternative was also recently used in an interventional study aiming to reverse the functional decline associated with acute hospitalization in very old patients [46].

### Strengths and limitations

The strengths of this study are its large sample size, its broad inclusion criteria, the use of accelerometers to assess PA and the large number of PA hours recorded. Regarding sample size, this study is the largest when compared to other studies in Europe [12, 13, 16–18, 41], the USA [20, 21, 32] and Australia [14, 19]. Regarding inclusion criteria, and contrary to previous studies [12, 32, 47, 48], we included patients with cognitive impairment/confusion as they are at increased risk of post-hospitalization functional decline [28, 39]. Finally, PA was assessed using accelerometers, which are considered superior to pedometers [49] and allow data analysis using different algorithms.

This study also has some limitations. First, the study was conducted in a single university hospital, which might limit generalizability, as patients attending a university hospital may present more comorbidities or more severe diseases than patients attending a general hospital. Hence, it would be important that this type of study be implemented in other settings. Second, some recordings were very short (< 24 h) and could not be used, or had a high percentage of accelerometer non-wear time. Still, our findings are similar to another study that used the same methodology and included all available data, and findings were similar when patients with a high non-wear time were excluded (supplemental Tables 2 and 3). Third, patients were free to choose on which side they wore the accelerometer; this might lead to differences, as the dominant hand was not systematically used. Still, a previous study [24] showed no difference in physical activity measurements when comparing dominant to non-dominant wrist and, similarly, no differences were found in our study (supplementary Table 5). Finally, our approach did not allow differentiating between PA in bed or elsewhere. Further studies could implement PA assessment by combining accelerometry with manual or automatic recording of location.

## Conclusion

Older hospitalized patients are physically active only 10% of daily time and concentrate their PA around eating periods. Whether a Barthel Index below 95 prior to inclusion may be used to identify patients at risk of physical inactivity during hospital stay remains to be proven.

## Supplementary information

**Additional file 1: Figure S1.** Flow diagram.

**Additional file 2: Figure S2.** examples of accelerometry graphs of a physically active and inactive patient.

**Additional file 3: Figure S3.** examples of vector magnitude distribution of a physically active and inactive patient.

**Additional file 4: Table S1.** characteristics of patients who accepted and did not accept to participate in the NEXT-STEP study, Lausanne, Switzerland. **Table S2.** Physical activity during the day according to gender, only participants with less than 20% accelerometry non-wear time, NEXT-STEP study, Lausanne, Switzerland. **Table S3.** Baseline characteristics of patients according to physical activity level, only participants with less than 20% accelerometry non-wear time, NEXT-STEP study, Lausanne, Switzerland. Physically active subjects were defined as being in the highest quartile of time spent in non-sedentary activities (definition 1) or as spending at least 20 minutes of moderate physical activity per day (definition 2). **Table S4.** Baseline characteristics of hospitalized elderly patients according to physical activity levels, NEXT-STEP study, Lausanne, Switzerland. Physically active patients were defined as being in the highest tertile (definition 3) or the highest quintile (definition 4) of time spent in non-sedentary activities. **Table S5.** Physical activity according to the position of the accelerometer, NEXT-STEP study, Lausanne, Switzerland.

## Data Availability

The datasets generated and/or analysed during the current study are not publicly available due to absence of consent from the participants and the Ethics Committee, but are available from the corresponding author on reasonable request. To preserve the participant’s identity, only metadata can be made available.

## Abbreviations

PA: Physical activity
IQR: Interquartile range
BASEC: Business Administration System for Ethics Committees
CER-VD: The Ethics committee of canton Vaud
i.e.: In other words
e.g.: For example
ADL: Activities of daily living
OR: Odds ratio
CI: Confidence interval
BMI: Body mass index
