# Supplementary material for: Accelerometry assessed physical activity of older adults hospitalized with acute medical illness - an observational study
Source: BMC Geriatr. 2020 Oct 2;20:382. doi: 10.1186/s12877-020-01763-w (PMC7532621; doi:10.1186/s12877-020-01763-w)
Supplement: Supplementary file 1 — Additional file 1: Figure S1. Flow diagram. [file 12877_2020_1763_MOESM1_ESM.pptx]

## Slide 1
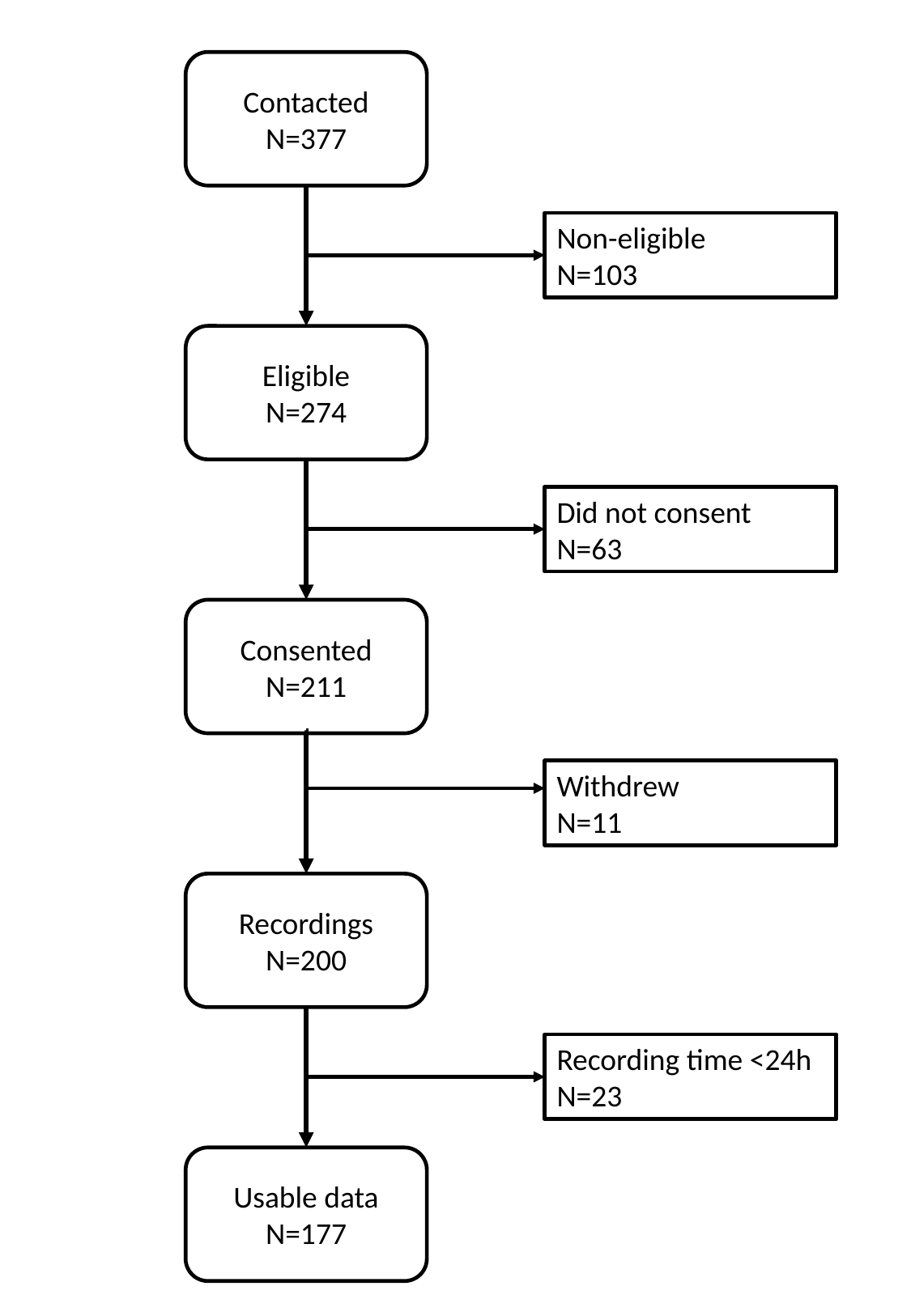

Contacted
N=377
Non-eligible
N=103
Eligible
N=274
Did not consent
N=63
Consented
N=211
Withdrew
N=11
Recordings
N=200
Recording time <24h
N=23
Usable data
N=177
