# Supplementary figures and images for: Accelerometry assessed physical activity of older adults hospitalized with acute medical illness - an observational study
Source: BMC Geriatr. 2020 Oct 2;20:382. doi: 10.1186/s12877-020-01763-w (PMC7532621; doi:10.1186/s12877-020-01763-w)

## Slide 1
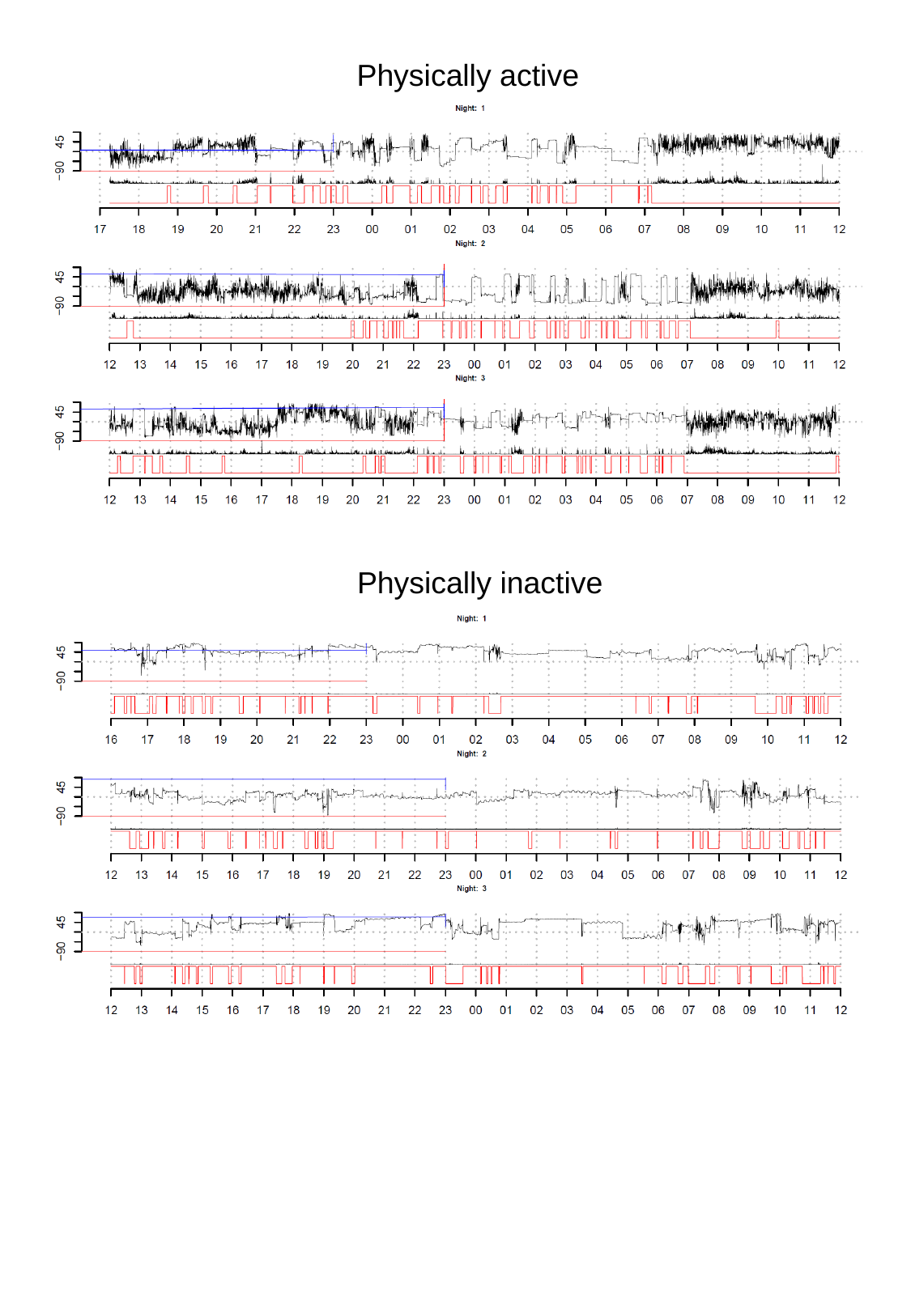

Physically active
Physically inactive

Supplement: Supplementary file 2 — Additional file 2: Figure S2. examples of accelerometry graphs of a physically active and inactive patient. [file 12877_2020_1763_MOESM2_ESM.pptx]

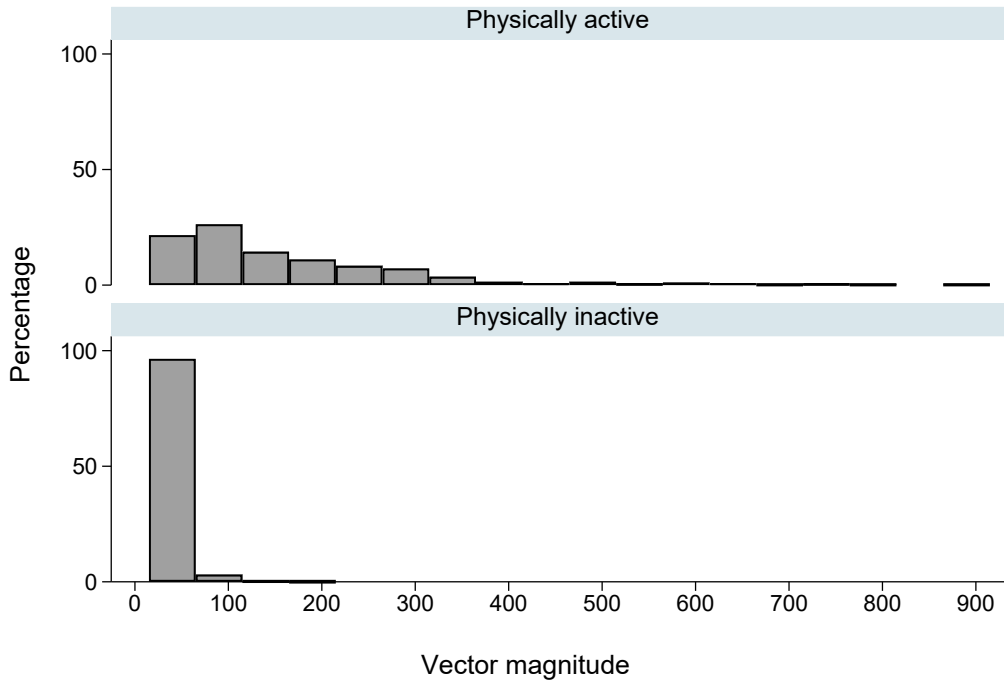

Supplement: Supplementary file 3 — Additional file 3: Figure S3. examples of vector magnitude distribution of a physically active and inactive patient. [file 12877_2020_1763_MOESM3_ESM.pdf]
