# Supplementary material for: Accelerometry assessed physical activity of older adults hospitalized with acute medical illness - an observational study
Source: BMC Geriatr. 2020 Oct 2;20:382. doi: 10.1186/s12877-020-01763-w (PMC7532621; doi:10.1186/s12877-020-01763-w)
Supplement: Supplementary file 4 — Additional file 4: Table S1. characteristics of patients who accepted and did not accept to participate in the NEXT-STEP study, Lausanne, Switzerland. Table S2. Physical activity during the day according to gender, only participants with less than 20% accelerometry non-wear time, NEXT-STEP study, Lausanne, Switzerland. Table S3. Baseline characteristics of patients according to physical activity level, only participants with less than 20% accelerometry non-wear time, NEXT-STEP study, Lausanne, Switzerland. Physically active subjects were defined as being in the highest quartile of time spent in non-sedentary activities (definition 1) or as spending at least 20 minutes of moderate physical activity per day (definition 2). Table S4. Baseline characteristics of hospitalized elderly patients according to physical activity levels, NEXT-STEP study, Lausanne, Switzerland. Physically active patients were defined as being in the highest tertile (definition 3) or the highest quintile (definition 4) of time spent in non-sedentary activities. Table S5. Physical activity according to the position of the accelerometer, NEXT-STEP study, Lausanne, Switzerland. [file 12877_2020_1763_MOESM4_ESM.docx]

**SUPPLEMENTAL TABLES**

**Supplemental table 1**: characteristics of patients who accepted and did not accept to participate in the NEXT-STEP study, Lausanne, Switzerland.

|  | **Yes** | **No** | **P-value** |
| --- | --- | --- | --- |
| N | 211 | 63 |  |
| Women (%) | 89 (42.2) | 38 (60.3) | 0.011 |
| Swiss nationality (%) | 200 (94.8) | 58 (92.1) | 0.419 |
| Clinical data upon admission (%) |  |  |  |
| Gait problems/fall | 54 (25.6) | 14 (22.2) | 0.587 |
| General state alteration | 71 (33.7) | 29 (46.0) | 0.073 |
| Dyspnea | 59 (28.0) | 19 (30.2) | 0.735 |
| Musculoskeletal pain | 9 (4.3) | 1 (1.6) | 0.463 § |
| Fever | 26 (12.3) | 5 (7.9) | 0.335 |
| Diarrhea / vomiting | 20 (9.5) | 6 (9.5) | 0.991 |

Results are expressed as number of patients (column percentage). Statistical analysis by chi-square or Fisher’s exact test (§).

**Supplemental table 2**: Physical activity during the day according to gender, only participants with less than 20% accelerometry non-wear time, NEXT-STEP study, Lausanne, Switzerland.

|  | **Men** | **Women** | **P-value** |
| --- | --- | --- | --- |
| Number of patients | 98 | 65 |  |
| Inactivity (min) |  |  |  |
| Minutes / day | 612 [510 - 651] | 598 [502 - 653] | 0.644 |
| % of daily time | 90.7 [84.8 - 94.3] | 90.4 [84.5 - 94.1] | 0.801 |
| Light activity |  |  |  |
| Minutes / day | 55 [32 - 87] | 67 [27 - 96] | 0.877 |
| % of daily time | 8.9 [5.3 - 14.4] | 9.3 [5.7 - 14.1] | 0.776 |
| Moderate activity |  |  |  |
| Minutes / day | 2 [1 - 8] | 2 [1 - 8] | 0.855 |
| % of daily time | 0.4 [0.2 - 1.2] | 0.4 [0.1 - 1.3] | 1.000 |
| Any physical activity |  |  |  |
| Minutes / day | 57 [33 - 93] | 68 [30 - 101] | 0.904 |
| % of daily time | 9.4 [5.6 - 15.1] | 9.6 [6.0 - 15.5] | 0.801 |

Results are expressed as median [interquartile range]. Between-group comparisons using Kruskal-Wallis test.

**Supplemental table 3**: Baseline characteristics of patients according to physical activity level, only participants with less than 20% accelerometry non-wear time, NEXT-STEP study, Lausanne, Switzerland. Physically active subjects were defined as being in the highest quartile of time spent in non-sedentary activities (definition 1) or as spending at least 20 minutes of moderate physical activity per day (definition 2).

|  | **Definition 1** | | | **Definition 2** | | |
| --- | --- | --- | --- | --- | --- | --- |
|  | **Inactive** | **Active** | **P-value** | **Inactive** | **Active** | **P-value** |
| Number of patients | 121 | 42 |  | 150 | 13 |  |
| **Characteristics** |  |  |  |  |  |  |
| Women | 48 (39.7) | 17 (40.5) | 0.927 | 59 (39.3) | 6 (46.2) | 0.630 |
| Age (years) | 81.4 ± 8.4 | 80.4 ± 8.4 | 0.507 | 81.2 ± 8.5 | 79.5 ± 7.1 | 0.486 |
| Body mass index (kg/m^2^) ǂ | 24.8 ± 4.6 | 24.5 ± 4.8 | 0.766 | 24.8 ± 4.7 | 23.3 ± 3.7 | 0.295 |
| Depressive disorders | 20 (16.5) | 4 (9.5) | 0.322‡ | 22 (14.7) | 2 (15.4) | 1.000‡ |
| Urinary/fecal incontinence | 43 (35.5) | 15 (35.7) | 0.984 | 54 (36.0) | 4 (30.8) | 0.773‡ |
| Hearing loss/vision issues | 46 (38.0) | 18 (42.9) | 0.580 | 59 (39.3) | 5 (38.5) | 0.951 |
| **Anamnesis** |  |  |  |  |  |  |
| Walking aids 2 weeks before admission | 67 (55.4) | 16 (38.1) | 0.054 | 80 (53.3) | 3 (23.1) | 0.045‡ |
| History of falls during the year before admission | 38 (31.4) | 14 (33.3) | 0.817 | 50 (33.3) | 2 (15.4) | 0.229‡ |
| Reason for admission associated with functional decline § | 67 (55.4) | 17 (40.5) | 0.096 | 82 (54.7) | 2 (15.4) | 0.008‡ |
| **Status upon inclusion** |  |  |  |  |  |  |
| Cognitive impairment/confusion | 34 (28.1) | 12 (28.6) | 0.953 | 46 (30.7) | 0 (0) | 0.020‡ |
| Sedative drugs | 16 (13.3) | 9 (21.4) | 0.211 | 23 (15.4) | 2 (15.4) | 1.000‡ |
| Barthel Index | 87.9 ± 16.2 | 92.7 ± 12.3 | 0.086 | 88.4 ± 15.9 | 97.2 ± 3.8 | 0.048 |
| Braden score |  |  | 0.088 |  |  | 0.161 |
| > 18 | 54 (46.6) | 26 (61.9) |  | 71 (49.0) | 9 (69.2) |  |
| ≤ 18 | 62 (53.5) | 16 (38.1) |  | 74 (51.0) | 4 (30.8) |  |
| Medical equipment † | 30 (24.8) | 4 (9.5) | 0.046‡ | 32 (21.3) | 2 (15.4) | 1.000‡ |
| Isolation precautions | 2 (1.7) | 3 (7.1) | 0.108‡ | 4 (2.7) | 1 (7.7) | 0.344‡ |
| Prescription of physiotherapy | 82 (67.8) | 24 (57.1) | 0.213 | 100 (66.7) | 6 (46.2) | 0.137 |
| Charlson comorbidity index | 4 [2 - 6] | 4 [2 - 6] | 0.491¶ | 4 [2 - 6] | 3 [2 - 7] | 0.642¶ |
| Number of comorbidities | 2 [1 - 4] | 2 [1 - 3] | 0.634¶ | 2 [1 - 4] | 2 [1 - 3] | 0.561¶ |

Results are expressed as mean ± SD or as median [interquartile range] for continuous variables and as number of participants (percentage) for categorical variables. ǂ, 148 participants. §: gait problems/ fall, general state alteration, musculoskeletal pain, neurological deficit. †: urinary catheter or oxygen therapy. Between-group comparisons using student’s t-test or Kruskal-Wallis test (¶) for continuous variables and chi-square or Fisher’s exact test (‡) for categorical variables.

**Supplementary table 4**: Baseline characteristics of hospitalized elderly patients according to physical activity levels, NEXT-STEP study, Lausanne, Switzerland. Physically active patients were defined as being in the highest tertile (definition 3) or the highest quintile (definition 4) of time spent in non-sedentary activities.

|  | **Definition 3** | | | **Definition 4** | | |
| --- | --- | --- | --- | --- | --- | --- |
|  | **Inactive** | **Active** | **P-value** | **Inactive** | **Active** | **P-value** |
| Number of patients | 118 | 59 |  | 141 | 36 |  |
| **Characteristics** |  |  |  |  |  |  |
| Women | 45 (38.1) | 26 (44.1) | 0.448 | 55 (39.0) | 16 (44.4) | 0.552 |
| Age (years) | 82.1 ± 8.6 | 79.6 ± 7.9 | 0.060 | 81.8 ± 8.4 | 79.1 ± 8.3 | 0.081 |
| Body mass index (kg/m^2^) ǂ | 24.6 ± 4.7 | 24.7 ± 4.6 | 0.969 | 24.7 ± 4.7 | 24.7 ± 4.7 | 0.981 |
| Depressive disorders | 19 (16.1) | 7 (11.9) | 0.453 | 21 (14.9) | 5 (13.9) | 0.879 |
| Urinary/fecal incontinence | 41 (34.8) | 19 (32.2) | 0.736 | 51 (36.2) | 9 (25) | 0.206 |
| Hearing loss/vision issues | 49 (41.5) | 21 (35.6) | 0.447 | 55 (39.0) | 15 (41.7) | 0.771 |
| **Anamnesis** |  |  |  |  |  |  |
| Walking aids 2 weeks before admission | 66 (55.9) | 24 (40.7) | 0.056 | 77 (54.6) | 13 (36.1) | 0.048 |
| History of falls during the year before admission | 36 (30.5) | 18 (30.5) | 1.000 | 44 (31.2) | 10 (27.8) | 0.690 |
| Reason for admission associated with functional decline § | 67 (56.8) | 26 (44.1) | 0.110 | 78 (55.3) | 15 (41.7) | 0.143 |
| **Status upon inclusion** |  |  |  |  |  |  |
| Cognitive impairment/confusion | 38 (32.2) | 14 (23.7) | 0.243 | 47 (33.3) | 5 (13.9) | 0.022 |
| Sedative drugs | 15 (12.8) | 12 (20.3) | 0.191 | 19 (13.6) | 8 (22.2) | 0.199 |
| Barthel Index | 87.3 ± 16.6 | 92.2 ± 13.6 | 0.049 | 87.1 ± 17 | 95.9 ± 6.1 | 0.003 |
| Braden score |  |  | 0.055 |  |  | 0.058 |
| > 18 | 53 (47.3) | 37 (62.7) |  | 66 (48.9) | 24 (66.7) |  |
| ≤ 18 | 59 (52.7) | 22 (37.3) |  | 69 (51.1) | 12 (33.3) |  |
| Medical equipment † | 30 (25.4) | 6 (10.2) | 0.017 | 32 (22.7) | 4 (11.1) | 0.165‡ |
| Isolation precautions | 2 (1.7) | 4 (6.8) | 0.078‡ | 5 (3.6) | 1 (2.8) | 1.000‡ |
| Prescription of physiotherapy | 83 (70.3) | 30 (50.9) | 0.011 | 95 (67.4) | 18 (50.0) | 0.053 |
| Charlson comorbidity index | 4 [2 - 6] | 4 [2 - 6] | 0.406¶ | 4 [2 - 6] | 4 [2 - 7] | 0.924¶ |
| Number of comorbidities | 3 [2 - 4] | 2 [1 - 3] | 0.037¶ | 2 [1 - 4] | 2 [1 - 3] | 0.450¶ |

Results are expressed as mean ± SD or as median [interquartile range] for continuous variables and as number of participants (percentage) for categorical variables. ǂ, 99 men and 63 women. §: gait problems/ fall, general state alteration, musculoskeletal pain, neurological deficit. †: urinary catheter or oxygen therapy. Between-group comparisons using student’s t-test or Kruskal-Wallis test (¶) for continuous variables and chi-square or Fisher’s exact test (‡) for categorical variables.

**Supplemental table 5**: Physical activity according to the position of the accelerometer, NEXT-STEP study, Lausanne, Switzerland.

|  | **Dominant hand** | **Non-dominant hand** | **P-value** |
| --- | --- | --- | --- |
| Number of patients | 107 | 70 |  |
| Inactivity (min) |  |  |  |
| Minutes / day | 623 [540 - 666] | 591 [469 - 661] | 0.211 |
| % of daily time | 90.7 [84.5 - 95.2] | 89.9 [85.6 - 93.2] | 0.417 |
| Light activity |  |  |  |
| Minutes / day | 63 [28 - 97] | 64 [37 - 103] | 0.579 |
| % of daily time | 8.8 [4.6 - 14.4] | 9.5 [6.3 - 13.1] | 0.369 |
| Moderate activity |  |  |  |
| Minutes / day | 2 [1 - 9] | 2 [1 - 9] | 0.952 |
| % of daily time | 0.4 [0.1 - 1.3] | 0.4 [0.2 - 1.2] | 0.878 |
| Any physical activity |  |  |  |
| Minutes / day | 64 [30 - 105] | 69 [38 - 108] | 0.621 |
| % of daily time | 9.3 [4.8 - 15.5] | 10.1 [6.7 - 14.4] | 0.423 |

Results are expressed as median [interquartile range]. Between-group comparisons using Kruskal-Wallis test.

**Multivariable model**

The stepwise logistic regression model to assess the variables significantly and independently related with being active was the following (Stata syntax):

stepwise, pe(0.05): logistic Quant_4 AUXTOOL BI_admi_catb MEDEQUIP PHYSIO comorbid COGNIMPAI

stepwise, pe(0.05): logistic Active AUXTOOL BI_admi_catb MEDEQUIP PHYSIO comorbid

Where:

- Quant_4: variable coded 0 (no) 1 (yes) defining being physically active according to definition 1 (being in the highest quartile of time spent in non-sedentary activities)
- Active: variable coded 0 (no) 1 (yes) defining being physically active according to definition 2 (spending at least 20 minutes of moderate physical activity per day).
- AUXTOOL: walking aids 2 weeks before admission, coded 0 (no) 1 (yes)
- BI_admi_catb: categories of the Barthel index, coded 0 (Slight/none) 1 (moderate) 2 (severe)
- MEDEQUIP: medical equipment (urinary catheter or oxygen therapy), coded 0 (no) 1 (yes)
- PHYSIO: prescription of physiotherapy, coded 0 (no) 1 (yes)
- Comorbid: number of comorbidities (continuous)
- COGNIMPAI: cognitive impairment/confusion, coded 0 (no) 1 (yes)

Note: due to estimability issues (cells with zero values) it was not possible to include variable COGNIMPAI in the stepwise analysis of Active.
